# On the Treatment of Remittent and Intermittent Fever by Quinine in the West Indies

**Published:** 1849-03

**Authors:** J. Davy


*On the Treatment of Remittent and Intermittent Fever by Quinine in the West Indies.* By J. Davy, M.D.—Dr. Davy says that of 165 cases of remittent fever treated in the hospital at Barbadoes, in the year 1847, only two proved fatal. Similar success has attended the British practice in the West Indies. He says—

In each station in which remittent fever has occurred, quinine, used so as to produce "cinchonism," appears to have been relied on, and is the chief remedial means employed. In Demerara, it has been commonly used without calomel; but aided by aperients, and when there has been irritability of the stomach, by a small dose of the solution of muriate of morphia, (15 drops), with a blister to the epigastrium. Staff-Surgeon Millar, principal medical officer in British Guiana, in his Quarterly Report, (which I would recommend to the attention of medical officers, when they have an opportunity to read it), states: "It requires, on an average, from twenty-five to thirty-five grains of the sulphate of quinine to saturate the system;" (that is, to produce cinchonism;) but this quantity must be given within the space of a few hours—four or five grains every hour, in solution, have been found sufficient, in a few hours, to effect it. If small doses are only given at long intervals, it will have no perceptible effect, long after the quantity mentioned has been given, and will, in all probability, prove so much valuable medicine thrown away. When dulness of hearing and ringing in the ears come on, no more of the medicine need be given; the patient will then be fonnd in a state of apyrexia, and the transition complete and striking.

When quinine is taken by an adult, to the extent of thirty or forty grains, it produces certain cerebral symptoms, the constituents of which are ringing noise in the ears, and more or less deafness.

This set of symptoms, where there is no idiosyncracy, indicates the saturation of the system by the medicine, as ptyalism does mercury, and may be conveniently known by the name of *cinchonism*.

Rare instances occur in which hyper-cinchonism is induced by a very few grains of quinine, accompanied by many nervous symptoms, and formication so severe as to proscribe the use of the remedy. In some—and this may occur in cases which had hitherto been normal—cinchonism has not been induced till after the administration of 72 grains of quinine.

Cinchonism is not peculiar to quinine: by other vegetable febrifuges, such as salicine, angustura bark, and biberine, cinchonism can be induced, but not with the same certainty as by quinine, neither in the same uniform series of phenomena, neither with the same harmlessness.

Cinchonism seldom lasts longer than twenty-four hours, except in some cases of anemia, in which the writer has known it continue upwards of a week.

Quinine has been prescribed by the writer to patients of both sexes and all ages, and where ascertainable, almost invariably to cinchonism, during thirteen years, and probably to the extent of several thousand ounces of the sulphate; and during that time he has seen no case of danger from its effects, with the exception of three or four cases of imputed abortion.

To many the muffled ears of cinchonism is not even disagreeable. Cinchonism is capable of superseding and surpassing that excited condition of the circulation and animal heat known as fever, except when depending on anemia, as symptomatic of inflammation, or its effects.

Quinine is purely a febrifuge: instead of being a tonic or stomachic, it generally induces anorexia, and a relaxed and macerated state of the skin, some tremulousness, and in many cases slight aphonia.

As a febrifuge, the full efficacy of quinine is seldom obtained, unless pushed to cinchonism. Cinchonism is therefore the test and criterion in practice of the full and sufficient use of quinine. It is probable that the protective influence of quinine against fever seldom lasts longer than the manifestation of cinchonism. The ordinary headache of fever does not contra-indicate the use of quinine.

The power of quinine seems to be to cut off the connexion between local irritation and constitutional excitement, to disturb and break the series of morbid elaborations set up in some specific fevers, which terminate, for the most part, in contamination of the blood and loss of vital cohesion of the capillaries. In intermittent fever it is antidotal.

Quinine is of little efficacy in intermittent fever, when exhibited during the paroxysm.

Quinine is of no efficacy in the last stages of continued or remittent fever, where the vascular and thermal excitement have been succeeded by organic lesion or contamination of the blood. It should be given, as is well known, in the intermission of intermittent fever, and in the formative, or in the first stage of continued, remittent, or yellow fever.

The use of quinine against relapses of intermittent fever, whether the disease has been primary or secondary, is one of its most valuable applications.

In using quinine against the paroxysms of intermittent fever, hourly doses of three grains, till twelve doses be given, is the best mode of saturating the system with the remedy. If, however, the disease be a quotidian, with short intermission, six-grain doses hourly, till six doses be given, will be judicious practice.

In the other fevers where quinine is eligible, and the remedy is prescribed during the existence of febrile excitement, the dose, to be efficacious, must be large, and the impression on the disease sudden and overwhelming.

An auxiliary, too, is also required in such cases: twenty-four grains of quinine and twenty grains of calomel, in one dose, is the most powerful resolvent of fever. One or two such doses, with an interval of six hours, and followed by a castor oil purgative, are generally sufficient; but I have prescribed six such doses with efficacy, and I recollect no instance of ptyalism occurring when this treatment was required and adopted, and sometimes there was but mild cinchonism. An intolerance of quinine, or early and intense cinchonism in such cases, is one of the worst prognostics.

In the treatment of simple intermittent fever, or its relapses, calomel is rarely, if ever prescribed by the writer. Sulphate and carbonate of magnesia mixture, or sulphate of magnesia and tartrate of antimony mixture, as a purgative during the hot stage, (if needed), or fifteen drops of solution of acetate of morphine, with a drachm of sweet spirits of nitre, if there is much suffering from muscular pains, headache, or emesis and retching, will speedily relieve the paroxysm; and followed by quinine in combination with purgative doses of rhubarb, will fulfil all the indications for the intermission.

But when a European or North American, probably not long from a cold climate, and during the prevalence of malignant disease, is attacked by fever, and shows to the quick and practiced eye alarming indications, no fear of the injurious after-effects of the mercurial will have weight to withhold the resolvent dose of calomel and quinine. In cases threatening danger to life only need it be used, and I know of no instance wherein the slightest untoward result has been experienced from its use.

The combination of quinine with tartar emetic in pneumonic and bronchitic complications of intermittent is eminently successful. The forces which disturb the remedial power of quinine in fever are chiefly inflammatory and congestive complications, or a loaded condition of the alimentary canal. These must be obviated by appropriate treatment, and the disease rendered as simple or idiopathic as possible, concurrent with the use of quinine. Thus arteriotomy may frequently be required in continued, remittent, or yellow fever; and in intermittent, with tenderness over the spleen, a blister may be required, as an auxiliary to cinchonism.

There is a form of continued, or irregular remittent fever, occurring chiefly in children or adolescents, in which generally no local cause can be discovered, but which is often imputed to worms; but give what anthelmintics you will, no worms may be passed; hence here they are popularly called "stubborn worms." This fever may continue for a week or a fortnight without any contamination of the blood or loss of vital cohesion, and probably depends on intestinal irritation. Danger in these cases chiefly arises from the supervention of some lesion, induced by the long-continued and excessive heat and violent action of the heart, or sympathetic irritation of the brain. In these cases I use quinine, with immediate and signal efficacy, in the following manner:

The patient is put into a bath, and the cold affusion is applied till the pulse becomes small, and nearly extinct, at the wrist, and the skin cold. He then, while in the bath, gets his dose of quinine, (two or three grains) and is returned to bed without being dried. The bath and the dose of quinine are continued hourly as long as the skin persists warm, when the hourly dose of quinine is due. After five or six baths the skin generally becomes permanently cool, and then the quinine is pushed on to cinchonism, alone, and without the bath. This mode of making an intermission in a continued fever I have never found attended with unpleasant or dangerous consequences, and it will generally subdue the fever after every other method has been tried in vain.

In fever of doubtful origin, and where latent inflammation is suspected, I have frequently used a small cantharides blister as a test; in fact, I never like to pass the blistered surface of a patient without inspecting it, its revelations are often so interesting and important. If, instead of the usual vesication of thin serum and cuticle, the vesication is a bladder of fibrinous coagulum, or suety in consistence, inflammatory action is going on, probably in the neighborhood of the part, and tartar emetic and such like combinations are indicated.

Relapses in intermittents have their determinate periods, the day from the last attack being generally some multiple of seven.

The usual day of relapse among the acclimatized of this colony is the fourteenth or twenty-eighth.

After one or two relapses, the law of each individual case can be ascertained by each patient.

The prophylactic which I have adopted with great success, and in my own person first, many years ago, is as follows:

Two days before the anticipated relapse, three grains of quinine, to be taken thrice daily for four days; after a similar relapse interval, the quinine to be again taken in the same manner; and so on, repeated three or four times successively. The disease is eradicated completely by thus baffling the relapse.—*London Lancet.*

